# 

**Published:** 1877-08

**Authors:** 


### ANNOUNCEMENTS FOR THE MONTH.

Societies—Mondays, August 6 and 20, Chicago Medical Society.

Clinics—Saturdays, at Rush College, 2 P. M. Surgical, Prof. Gunn. Tuesdays, at Chicago Medical College, 9 A. M. Surgical, Prof. Hyde.

Lectures—Mondays, 4 P. M., at Cook Co. Hospital Necroscopy Theatre. On Pathological Anatomy, Prof. I. N. Danforth.

